# Cerebral arteriovenous malformations in the era of embolization for angiographic cure: a single-center experience in Egypt

**DOI:** 10.1186/s41983-018-0003-5

**Published:** 2018-05-03

**Authors:** Mohamed K Elewa

**Affiliations:** Neurology Department, Ain Shams University, 38 El-Abbasia, Cairo, 11566 Egypt

**Keywords:** Arteriovenous malformation, Embolization, Nidus, Onyx, Outcome

## Abstract

**Background:**

Embolization for cerebral arteriovenous malformations (AVMs) has evolved in the last decade with evolution in both equipment and material. Embolization targets have expanded to include angiographic cure.

**Methods:**

To discuss the technical and management outcomes of our first cerebral AVM case series treated with embolization. The clinical, angiographic, treatment, and outcome variables of consecutive cerebral arteriovenous malformation cases, treated with curative embolization, between January 2011 and June 2017 in one regional center, were retrospectively analyzed.

**Results:**

In 21 patients, 21 AVMs were identified, and 13 patients (61.9%) were males. The mean of the age was 34.24 ± 12.99. Fifteen patients (71.4%) had a history of intracranial hemorrhage, and 10 (47.6%) patients had seizures. Sixteen patients (76.2%) were at grade 1 of modified Rankin Scale (mRS) at admission. The median for modal Spetzler-Martin grade was 2. The average number of arterial feeders was 3. Direct arteriovenous fistulas were found in 4 cases (19.0%). Venous aneurysms were found in 4 cases (19.0%). Seventeen AVMs (80.9%) were considered high bleeding risk lesions. Forty-three embolization sessions were done. Early hemorrhage occurred in 3 sessions (7.0%). Vessel perforation occurred 1 session (2.3%). Poor outcome occurred in 1 patient that was discharged at grade 3 mRS. Angiographic cure was achieved in 9 patients (42.9%). The average size reduction was 65%.

**Conclusions:**

Onyx embolization could serve as a curative option for AVM treatment with accepted morbidity and mortality.

## Background

Over the past decades, the target of embolization has evolved, starting as preoperative embolization to facilitate AVM resection (Crowley et al. 2014). Although it is not approved yet by the US Food and Drug Administration (FDA), pre-radiosurgical embolization is performed to eliminate high-risk features or to reduce nidal size (Pierot et al. 2013a). With evolution in both materials [e.g., ethylene-vinyl alcohol co-polymer (EVOH)] and techniques, the indications for endovascular embolization of cerebral arteriovenous malformations (AVMs) have expanded to include curative embolization (Mounayer et al. 2007; Panagiotopoulos et al. 2009).

Despite the wide variety of the embolic agents that can be used, only few agents are commonly used [*n*-butyl cyanoacrylate (NBCA), ethylene-vinyl alcohol copolymer (EVOH) (Onyx) (Covidien/ev3, Plymoth, Massachusetts, USA), and, to a lesser extent, polyvinyl alcohol (PVA) particles] (Vaidya et al. 2008). Glue (NBCA) has several privileges, including the immediate complete permanent vascular occlusion. Unfortunately, it requires high degree of experience for its proper use. Several complications can occur, including catheter entrapment (Debrun et al. 1997), distal spread, and proximal reflux beyond the intended location of the injection. Onyx (EVOH) in comparison to NBCA is less thrombogenic with minimal inflammatory reaction, since there is no protein denaturation (Duffner et al. 2002; Vinters et al. 1985). The major advantage of Onyx is the non-adhesive nature, which allows longer injection time, with the ability to perform control angiography when needed. Despite the non-adhesive nature of Onyx, still there is a risk of catheter entrapment due to reflux around the catheter tip. This article reported our experience in the treatment of cerebral AVMs using Onyx mainly. Our policy was to use embolization as a curative option whenever possible.

## Methods

**Fig. 1:**
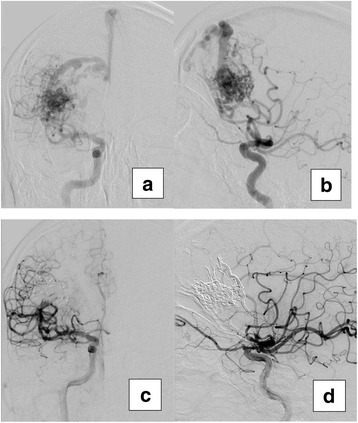
An elderly patient with a ruptured small right frontal brain AVM. **a** Frontal view of a right internal carotid angiogram shows multiple feeders from the middle cerebral artery and superficial venous drainage. **b** Lateral view. **c**, **d** Final result (complete occlusion) after embolization of 3 feeders

A retrospective review of all consecutive patients who had underwent endovascular embolization for cerebral AVM between January 2011 and June 2017 in Suez Insurance Hospital was performed. Twenty-one AVMs in 21 patients were included in the current study. All patients were fulfilling the following inclusion criteria: ruptured or unruptured AVM (either symptomatic or discovered accidently) with high bleeding risk due to small size (< 3 cm), deep venous drainage, and/or associated aneurysm (Fig. 1). Patients with recent intracranial hemorrhage were postponed for at least 1 month (hematomas may interfere with good angiographic assessment) (Pierot et al. 2013b). They were considered for neurosurgical opinion, in case of intraventricular hemorrhage or hemorrhage that cause mass effect; otherwise, a conservative therapy was implemented. None of those patients developed rebleeding with these conservative measures (Reitz et al. 2016).

All patients underwent clinical assessment and modified Rankin Scale (mRS) before and after all embolization sessions and during follow-ups, and before treatment, all AVMs were assessed by six-vessel digital subtraction cerebral angiography (DSCA) according to their eloquent site, nidus type, presence of fistulas, feeding arteries and draining veins, number of the main feeders, presence of aneurysms, and presence of venous stenosis. Spetzler-Martin grading (Spetzler and Martin 1986) was identified for all AVMs. Eloquent areas were identified according to Pollock and Flickinger’s (2002) definition: frontal and temporal (not invading the speech centers and motor strip) are non-eloquent; fronto-parietal, speech, parietal, occipital, intraventricular, corpus callosum, and cerebellar are intermediate; and basal ganglia, internal capsule, thalamic, and brain stem are considered eloquent. After the last embolization session, a follow-up angiography was performed 3 months later, except when other treatment modality is needed, follow-up DSA was performed after 1 month. A “poor” outcome was defined as a mRS score of 3–6 at discharge. Cure was defined as angiographic complete obliteration of the AVM nidus with disappearance of early veins.

### Technique of intervention and periprocedural management

All procedures were carried out under general anesthesia, without provocative testing (Amytal), via femoral puncture. Six-vessel DSCA was done for all patients. Systemic heparinization was done once the endovascular therapy was decided. Then, selective catheterization of the target vessel (either the internal carotid or the vertebral artery) was done using 6F guiding catheter (Guider Softip, Boston Scientific, Natick, MA, USA). Then, the AVM angio-architectural characteristics were studied. Using coaxial system [DMSO compatible microcatheter with detachable tip, Apollo (eV3, Inc., Irvine, California) or Sonic (Balt, Montmorency, France) and 0.008 micro-guidewire, Mirage (eV3, Inc., Irvine, California)], the pathway to the AVM was navigated, then one of the AVM feeders was selectively catheterized, and the tip of the microcatheter was placed as close as possible to the nidus (most distal part of the arterial contribution). Then, an angiographic series was taken through the microcatheter to study the AVM architecture, adjust the microcatheter position for safe reflux (before the detachment mark), and adjust the angiographic projection for better visualization of the proximal part of the draining veins. Micro-angiography was useful also in evaluation of the flow state before embolization. In normal follow state, we used Onyx 18. The Onyx vials were placed on a shaker for at least 20 min to obtain a homogeneous solution before use. Then, Onyx 18 and DMSO were prepared in two different 1-mL Leur-Lock syringes. The dead space of the microcatheter was filled with DMSO before the injection of Onyx. Onyx was injected slowly (0.1 mL/min) under free-flow condition and on subtracted fluoroscopy. The initial target was to form a cast of Onyx reflux, over a short distance of the catheter tip. In case of reflux, Onyx injection was stopped, and after 60 s, another slow injection was started under a new subtracted road map fluoroscopy. If there was forward expansion of Onyx, the injection was continued; if reflux occurs or there was expansion to veins, the injection was stopped again. The cycle was repeated patiently until there was no forward advancement into the nidus and reflux was repeatedly the result of the injection. When necessary, the same was repeated for other feeders. Towards complete occlusion of the AVM nidus, Onyx was progressively allowed to enter the veins but with frequent pauses to redirect Onyx into the remaining parts of the nidus, even in retrograde fashion to different arterial feeders. Even when angiography showed complete occlusion of the AVM, Onyx was injected to completely occlude the proximal part of the draining veins, until reflux into the feeders prevents further Onyx injection. Before removal of the microcatheter, Onyx was aspirated. Then, by applying slowly increasing and decreasing traction tension in cycles, the microcatheter can be released from the cast. For high-flow fistulas (NBCA), Histoacryl (B. Braun Melsungen AG, Melsungen, Germany) was used. We used highly concentrated glue diluted with lipiodol (ranged from 50 to 70%), injected in rapid short injection. The next embolization sessions were scheduled with at least 1-month intervals. After angiographic cure, a follow-up angiography was performed after 3 months. In case of AVM nidal remnants not suitable for embolization, patients were sent for radiosurgery.

Data were analyzed using Statistical Program for Social Science (SPSS) version 20.0. (SPSS, Chicago, IL). Quantitative data were expressed as mean ± standard deviation (SD). Qualitative data were expressed as frequency and percentage. The *χ*^2^ of significance was used to compare proportions between two qualitative features. In all tests, *P* value less than 0.05 was considered significant, *P* value less than 0.001 was considered highly significant, and *P* value greater than 0.05 was considered nonsignificant.

## Results

Patients were assigned to treatment between January 2011 and June 2017. Thirteen patients (61.9%) were males. The range of the age was 9–61 years, while the mean of the age was 34.24 ± 12.99. Hypertension was the most prevalent risk factor in 2 patients (9.5%). Fifteen patients (71.4%) had a history of intracranial hemorrhage, 10 patients (47.6%) had a history of seizures, and 4 patients (19.0%) were complaining of chronic headache. Regarding modified Rankin Scale, 16 patients (76.2%) were at grade 1, 4 patients (19.0%) were at grade 2, and 1 patient (4.8%) was at grade 3 (Table 1).

**Table 1:** Primary clinical assessment

	Total (*N* = 21)
Sex	
Females	8 (38.1%)
Males	13 (61.9%)
Age (years)	
Range [mean ± SD]*	9–61 [34.24 ± 12.99]
Hypertension	2 (9.5%)
Clinical presentation	
Intracranial hemorrhage	15 (71.4%)
Epilepsy	10 (47.6%)
Headache	4 (19.0%)
Others	0 (0.0%)
Modified Rankin Scale	
Score 1	16 (76.2%)
Score 2	4 (19.0%)
Score 3	1 (4.8%)

*SD standard deviation

**Fig. 2:**
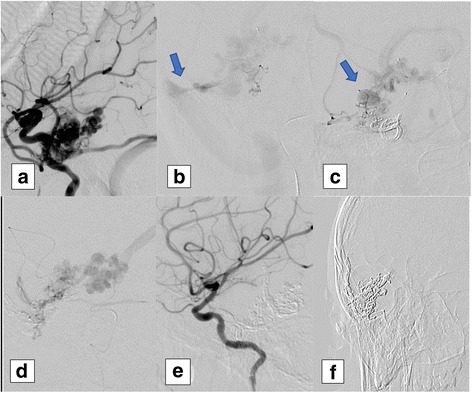
A pediatric case with unruptured right temporal brain AVM. **a** Lateral view of a right internal carotid angiogram shows multiple feeders from the middle cerebral artery and superficial and deep venous drainage. **b** shows the 1st feeder angiogram with venous stenosis (arrow), **c** shows the 2nd feeder angiogram with venous aneurysm (arrow), and **d** shows the 3rd feeder angiogram. **e** Final result (complete occlusion) after embolization of 3 feeders. **f** shows Onyx cast after complete occlusion of the AVM

Among 21 patients, 21 AVMs were identified. According to their sites, 9 AVMs (42.9%) were frontal, 7 AVMs (33.3%) were parietal, 3 AVMs (14.3%) were temporal, and 2 AVMs (9.5%) were occipital. The range of the total number of feeders was 1–8, while the median was 3. Four AVMs were associated with direct arteriovenous fistula. Four AVMs (19.0%) were associated with venous aneurysms. Thirteen AVMs (61.9%) were < 3 cm in size, 7 AVMs (33.3%) were > 3 and < 6 cm, and 1 AVM (4.8%) was > 6 cm. Fourteen AVMs (66.7%) were present in eloquent site. Five AVMs (23.8%) had deep venous drainage (not-exclusive) (Fig. 2). Regarding Spetzler-Martin grading, the median was 2. Seventeen AVMs (80.9%) were classified as high bleeding risk lesions (small AVMs, associated aneurysm, and/or deep venous drainage) (Table 2).

**Table 2:** Angiographic assessment

Angiographic assessment	Total (*N* = 21)
Site	
Frontal	9 (42.9%)
Parietal	7 (33.3%)
Temporal	3 (14.3%)
Occipital	2 (9.5%)
Total number of feeders	
Range [median (IQR)]*	1–8 [3 (3)]
Direct fistula	4 (19.0%)
Associated aneurysms	4 (19.0%) (venous)
Venous stenosis	3 (14.3%)
Size	
< 3 cm	13 (61.9%)
3–6 cm	7 (33.3%)
> 6 cm	1 (4.8%)
Eloquent site	14 (66.7%)
Deep venous drainage	5 (23.8%)
Spetzler-Martin grading	
Range [median (IQR)]	1–5 [2 (1)]
High bleeding risk	17 (80.9%)

*IQR interquartile ratio

Forty-three embolization sessions, with a total of 59 feeders in 21 AVMs, were embolized, in attempt to treat 21 AVMs in 21 patients. Early hemorrhage occurred in 3 sessions (7.0%) for 2 patients (9.5%); all were treated conservatively with good functional recovery. In 1 patient with recurrent early postoperative hemorrhage, venous outflow obstruction was assumed to be the cause of the recurrent event; in the other patient, no obvious cause was identified. Vessel perforation occurred once and was treated immediately intra-operatively with onyx with no adverse clinical consequences. At discharge, only 2 patients were at grade 3 mRS (one of them was already on grade 3 on admission). The average size reduction was 65% with a range of 5–100%. Angiographic cure was achieved in 9 patients (42.9%). The mean of the time to angiographic cure was 2.11 ± 1.45 months; the range was 0–4 months. The mean of the follow-up duration was 45.52 ± 20.27 months, while the range was 2–78 months (Table 3).

**Table 3:** Operative data and outcome

	Total number of sessions (*N* = 43)
Sessions per patient	
Range [median (IQR)]*	1–4 [2 (1)] sessions
Embolized feeders per session	
0	1 (2.3%)
1 feeder	25 (58.1%)
2 feeders	17 (39.5%)
Early hemorrhage	3 (7.0%)
Vessel perforation	1 (2.3%)
Thrombo-embolic events	0 (0.0%)
Late hemorrhage	0 (0.0%)
	Total number of patients (*N* = 21)
Modified Rankin Scale at discharge	
Score 1	14 (66.6%)
Score 2	5 (23.8%)
Score 3	2 (9.5%)
Size reduction	
% range [median (IQR)]	5–100 [65 (55)]
Angiographic cure	9/21 (42.9%)
Time to cure (months)	
Range [mean ± SD]^†^	0–4 [2.11 ± 1.45]
Follow-up duration (months)	
Range [mean ± SD]	2–78 [45.52 ± 20.27]

*IQR interquartile range

^†^*SD* standard deviation

In attempt to identify factors that were associated with angiographic cure, a comparison between totally occluded AVMs (angiographic cure) and partially occluded AVMs regarding the site, total number of feeders, size, eloquent site, and deep venous drainage was done. Only eloquent site of the AVM was found to be significantly associated with partial occlusion (Table 4).

**Table 4:** Comparison between totally occluded AVMs and partially occluded AVMs

	Partial occlusion (*N* = 12)	Total occlusion (*N* = 9)	*χ*^2^/*z*	*P* value
Site				
Frontal	4 (33.3%)	5 (55.6%)	3.662	0.330
Temporal	1 (8.3%)	2 (22.2%)		
Occipital	1 (8.3%)	1 (11.1%)		
Parietal	6 (50.0%)	1 (11.1%)		
Total number of feeders	1–8 [3 (2)]	1–5 [3 (3)]	− 0.146	0.884
Size				
Large	6 (50.0%)	7 (77.8%)	1.974	0.373
Medium	5 (41.7%)	2 (22.2%)		
Small	1 (8.3%)	0 (0.0%)		
Eloquent site	10 (83.3%)	4 (44.4%)	3.500	*0.041*
Deep venous drainage	3 (25.0%)	2 (22.2%)	0.022	0.882

## Discussion

The target of brain AVM treatment is to eliminate the risk of bleeding. This target could be achieved by complete occlusion of AVM. Various options are available (embolization, surgery, and radiosurgery) either alone or in combination. Embolization usually used as the first step in treatment plan, but in two different strategies. In the first strategy, the target is to achieve occlusion as much as possible before thinking in other options. The second strategy is to use embolization to prepare the patient to other modality of treatment. In the current study, we used the first strategy whenever possible.

A recent meta-analysis showed that the average cure rate for all NBCA-used studies was 13.7%. The cure rate increased to 24% in a subgroup analysis (387 patients) for studies conducted after the year 2000 (Elsenousi et al. 2016). Valavanis and Yaşargil (1998) embolized 7.7 feeders per AVM using NBCA with a cure rate of 40%. On the other hand, ethylene-vinyl alcohol copolymer (EVOH) (Onyx; eV3-Covedien, Irvine, California, USA) average cure rate was 30.5% in all studies, and 30.1% in the studies that did not use detachable microcatheters (Elsenousi et al. 2016a). The BRAVO trial was a European prospective multicenter trial to assess the safety and efficacy of Onyx. It showed 23.5% complete occlusion rate using onyx (Pierot et al. 2013b). Meanwhile, the range of complete occlusion rates in published case series was 8.3–53.9% (Pierot et al. 2013b). In the current study, complete occlusion was achieved in 9 patients (42.9%), this cure rate is similar to the previously quieted figures, and this high cure rate could be explained by the prior intention to completely occlude the lesion whenever possible, the use of Oynx with detachable tip catheters which permit longer injection time, and the use of staged approach. With case selection, van Rooij et al. (2012) reported achievement of cure for all the selected patient (24 patients) using Onyx without morbidity or mortality; also, de Castro-Afonso et al. (2016) reported (91.3%) cure rate in their selected AVM pediatric patients (23 patients) with (13%, 3 patients) procedure-related complications, yet without significant morbidity or mortality.

A new trend of embolization of brain AVMs with intent to angiographic cure was noticed. In some centers, curative embolization is offered to selected patients (van Rooij et al. 2012; de Castro-Afonso et al. 2016). This trend was encouraged by the expansion of the use of Onyx. This was reflected by higher cure rates of onyx compared with glue, but this was claimed to be associated with worse neurological outcomes. On the other hand, NBCA showed higher cure rates with time also, but without increase in poor neurological outcome. The assumption that the introduction of detachable microcatheter was associated with better neurological outcome was not supported by the results of meta-analysis. This may be explained by the fact that the use of the detachable microcatheters was associated with more aggressive Onyx embolization that may lead to venous occlusion and hemorrhagic complications (Elsenousi et al. 2016). It seems that the results of meta-analysis in this area should not be generalized, as it depends on operator experience which is variable, and cautious slow controlled injections that avoid the draining veins can minimize the procedural risk even in eloquent locations with longer injection time especially with the use of detachable tip microcatheters.

A meta-analysis showed that poor neurological outcomes for glue and Onyx embolization were 5.2 and 6.8%, respectively. When the results for patients embolized with Onyx using detachable catheters were excluded, poor outcome associated with Onyx embolization increased to 7.1% (Elsenousi et al. 2016). The BRAVO trial showed that Onyx embolization is associated with 4.3% mortality and 5.1% morbidity (Pierot et al. 2013b). The range of hemorrhagic complication in published case series was 4.0–12.2% while the range of mortality and morbidity was 0.0–3.2 and 3.5–15.5%, respectively (Pierot et al. 2013b). In the current study, early hemorrhage occurred in 3 sessions out of 43 sessions (7.0%) for 2 patients (9.5%). Only 2 patients (9.5%) were at grade 3 mRS at discharge (one of them was already on grade 3 on admission) which goes in line with the previously published data.

In our series, 17 AVMs (80.9%) were classified as high bleeding risk lesions. Thirteen AVMs (61.9%) were < 3 cm in size, 5 AVMs (23.8%) had deep venous drainage (not-exclusive), and 4 AVMs (19.0%) were associated with venous aneurysms. Crawford et al. (1986) described that 7% of the AVMs were associated with aneurysms and 75% of these aneurysms located on the major feeder vessels. AVM-associated aneurysms tend to present 10 years later than AVMs (Ondra et al. 1990). D’Aliberti et al. in their series found that the different types of AVM-associated aneurysms had different bleeding risk. The bleeding risk of the unrelated type and the remote flow-related type should be considered almost the same as any else unruptured aneurysm, where the INSUIA parameters were applicable. On the other hand, the adjacent flow-related type and the venous type carried higher bleeding risk (D’Aliberti et al. 2015). Choi et al. (2005) had suggested that AVMs with deep or infratentorial location, single or few draining veins, venous stenosis, and intranidal aneurysms may had increased risk of bleeding. Guo et al. (1995) found that small AVMs (< 3 cm) carried higher risk of bleeding. Meanwhile, deep venous drainage was found to be independent risk factor for AVM bleeding (Stapf et al. 2006). Although infratentorial AVM in adults account for only 7 to 15% of all brain AVMs, it was found to be an independent risk factor for bleeding; the bleeding rates were high up to 92% (Arnaout et al. 2009; Khaw et al. 2004; Westphal and Grzyska 2000).

The average age at presentation in this study was 34 years, which was in keeping with the previously quoted figure of 33 years (Ondra et al. 1990). In a recent meta-analysis for the outcome of embolization of cerebral AVMs, the mean for patients’ age was 35.8 and 35 years in the glue and Onyx groups, respectively (Elsenousi et al. 2016). AVMs were known to show male predominance, which went in the same direction with the current study findings [13 patients (61.9%) were males] (Ondra et al. 1990).

In the current series, 15 patients (71.4%) had a history of intracranial hemorrhage, 10 patients (47.6%) had a history of seizures, and 4 patients (19.0%) were complaining of chronic headache. In a meta-analysis, hemorrhage was the presenting symptom in 47% of patients in the glue and 41.5% of patients in the Onyx group. Epileptic seizures were the presenting symptom in 28% of the glue group and in 33% of the Onyx group (Elsenousi et al. 2016). Hemorrhagic presentation accounted for 50–61% of cases (Drake 1979; Perret and Nishioka 1966). AVM bleeding was associated with neurological deficit in 30–50% of cases only (Hetts et al. 2014). The high rate of bleeding as a presenting event in our series could be explained by the fact that 17 AVMs (80.9%) were classified as high bleeding risk lesions or due to small size of the sample. An association between age and the incidence of seizures was described by Crawford et al. (1986), with a seizure risk 44% in patients aged 10–19 years, 31% in patients aged 20–29 years, and 6% risk in patients aged 30–60 years.

Interventional treatment of AVMs is generally accepted; the annual risk of bleeding is 2–4%, which occurs over time, with high lifetime risk of bleeding that can result in morbidity or mortality (Young et al. 2015). The ARUBA (a randomized trial for unruptured brain arteriovenous malformation) has challenged this concept. The study concluded that medical treatment alone is better than interventional options, for prevention of stroke or death in patients with unruptured brain AVMs (Mohr et al. 2014). Despite the importance of the study, criticism to its design and follow-up duration undermined the value of its conclusion (Russin and Spetzler 2014). The poor outcome in the intervention arm of ARUBA trial is 30%, which is much higher than what was reported in a meta-analysis (5.2% for NBCA and 6.8% for EVOH) (Elsenousi et al. 2016); on the other hand, this poor outcome is also higher than the published rates of poor outcome for surgical resection of AVMs (0.34–2.2% in grades I–II) (Spetzler and Martin 1986; 2013). It was not clear how much of the poor outcome was related to surgical resection, embolization, or delayed hemorrhage from partially embolized AVM. On the other hand, a curative embolization with Onyx with high cure rates as recently published (van Rooij et al. 2012; de Castro-Afonso et al. 2016) that was associated with low morbidity and mortality (< 10%) may serve as a good treatment option. A low complication rate (< 10%) is lower than the complication rate of the medical arm of the ARUBA trial (Mohr et al. 2014). Six patients (28.57%) in our case series had unruptured AVM. Embolization treatment was considered for these cases due to the presence of high bleeding risk criteria of these AVMs.

In the current study, we used the staged approach in AVMs with multiple feeders. Despite that the value of this approach was debated, we thought it was associated with less hemorrhagic complications (Mounayer et al. 2007). This approach allowed smooth normalization of hemodynamics and prevented extensive venous thrombosis. The use of NBCA (glue) was limited to high flow fistulas in 4 patients (19.0%), where we thought it was more simple and effective method.

We compared between patients who achieved angiographic cure and patients who had partial occlusion. The comparison included the site, the total number of feeders, the size, the presence in eloquent site, and the presence of deep venous drainage. Only the presence of AVM in eloquent site was significantly associated with partial occlusion. This finding was logic, but we thought that larger sample size of study was needed to identify more factors that were associated with angiographic cure.

We think that curative embolization should be offered for selected cases based on certain criteria that need further research. For the cases that are not fulfilling these criteria, embolization should be offered as preparing step for other modality of treatment. For curative embolization, the AVM should be small sized (< 3 cm), supplied by one vascular territory, with feeders that can tolerate reflux up to 2–3 cm, with clear proximal parts of the draining veins, and not located in deep structure or brain stem (van Rooij et al. 2012).

## Conclusions

Onyx embolization could serve as a curative option with accepted morbidity and mortality. Understanding the angio-architectural characteristics of the treated AVM could help in better differentiation between lesions suitable for curative Onyx embolization, and those non-suitable for curative embolization, with fewer treated feeders and high occlusion rate. Good selection of intranidal catheter tip position with slow, controlled injections that avoid the draining veins can minimize the procedural risk even in eloquent locations.

Several limitations should be considered in the current study: the retrospective nature, the potential patient selection for treatment bias, the small size of the sample, the absence of other modalities of interventional treatment, and the lack of cases that were treated on emergency basis.

## References

[CR1] Arnaout OM, Gross BA, Eddleman CS, Bendok BR, Getch CC, Batjer HH (2009). Posterior fossa arteriovenous malformations. Neurosurg Focus.

[CR2] de Castro-Afonso LH, Nakiri GS, Oliveira RS, Santos MV, Santos ACD, Machado HR, Abud DG (2016). Curative embolization of pediatric intracranial arteriovenous malformations using Onyx: the role of new embolization techniques on patient outcomes. Neuroradiology.

[CR3] Choi JH, Mohr JP. Brain arteriovenous malformations in adults. Lancet Neurol. 2005;4(5):299–308. 10.1016/S1474-4422(05)70073-9 15847843

[CR4] Crawford PM, West CR, Chadwick DW, Shaw MD (1986). Arteriovenous malformations of the brain: natural history in unoperated patients. J Neurol Neurosurg Psychiatry.

[CR5] Crowley RW, Ducruet AF, McDougall CG, Albuquerque FC (2014). Endovascular advances for brain arteriovenous malformations. Neurosurgery.

[CR6] D'Aliberti G, Talamonti G, Cenzato M, La Camera A, Debernardi A, Valvassori L, Mariangela P, Nichelatti M (2015). Arterial and venous aneurysms associated with arteriovenous malformations. World Neurosurg.

[CR7] Debrun GM, Aletich VA, Shownkeen H, Ausman J (1997). Glued catheters during embolisation of brain AVMs with acrylic glue. Interv Neuroradiol.

[CR8] Drake CG (1979). Cerebral AVMs: considerations for and experience with surgical treatment in 166 cases. Clin Neurosurg.

[CR9] Duffner F, Ritz R, Bornemann A, Freudenstein D, Wiendl H, Siekmann R (2002). Combined therapy of cerebral arteriovenous malformations: histological differences between a non-adhesive liquid embolic agent and n-butyl 2-cyanoacrylate (NBCA). Clin Neuropathol.

[CR10] Elsenousi A, Aletich VA, Alaraj A. Neurological outcomes and cure rates of embolization of brain arteriovenous malformations with n-butyl cyanoacrylate or Onyx: a meta-analysis. J Neurointerv Surg. 2016;8(3):265–72. 10.1136/neurintsurg-2014-011427 25540177

[CR11] Guo WY, Pan DH, Liu RS, Chung WY, Shiau CY, Cheng SS (1995). Early irradiation effects observed on magnetic resonance imaging and angiography, and positron emission tomography for arteriovenous malformations treated by gamma knife radiosurgery. Stereotact Funct Neurosurg.

[CR12] Hetts SW, Cooke DL, Nelson J, Gupta N, Fullerton H, Amans MR (2014). Influence of patient age on angioarchitecture of brain arteriovenous malformations. AJNR Am J Neuroradiol.

[CR13] Khaw A, Mohr J, Sciacca R, Schumacher H, Hartmann A, Pile-Spellman J, et al. Association of infratentorial brain arteriovenous malformations with hemorrhage at initial presentation. Stroke. 2004;35:660–63. 10.1161/01.STR.0000117093.59726.F9 14752127

[CR14] Mohr JP, Parides MK, Stapf C, Moquete E, Moy CS, Overbey JR, et al and Investigators International ARUBA Medical management with or without interventional therapy for unruptured brain arteriovenous malformations (ARUBA): a multicentre, nonblinded, randomised trial. Lancet 2014 ; 383 (9917): 614–621. 10.1016/S0140-6736(13)62302-8 PMC4119885 24268105

[CR15] Morgan MK, Davidson AS, Koustais S, Simons M, Ritson EA. The failure of preoperative ethylene-vinyl alcohol copolymer embolization to improve outcome in arteriovenous malformation management: case series. J Neurosurg 2013;118:969–77. 10.3171/2012.11.JNS112064 23350776

[CR16] Mounayer C, Hammami N, Piotin M, Spelle L, Benndorf G, Kessler I, Moret J (2007). Nidal embolization of brain arteriovenous malformations using Onyx in 94 patients. Am J Neuroradiol.

[CR17] Ondra SL, Troupp H, George ED, Schwab K (1990). The natural history of symptomatic arteriovenous malformations of the brain: a 24-year follow-up assessment. J Neurosurg.

[CR18] Panagiotopoulos V, Gizewski E, Asgari S, Regel J, Forsting M, Wanke I (2009). Embolization of intracranial arteriovenous malformations with ethylene-vinyl alcohol copolymer (Onyx). AJNR Am J Neuroradiol.

[CR19] Perret G, Nishioka H (1966). Report on the cooperative study of intracranial aneurysms and subarachnoid hemorrhage: arteriovenous malformations. J Neurosurg.

[CR20] Pierot L, Cognard C, Herbreteau D, Fransen H, van Rooij WJ, Boccardi E (2013). Endovascular treatment of brain arteriovenous malformations using a liquid embolic agent: results of a prospective, multicentre study (BRAVO). Eur Radiol.

[CR21] Pierot L, Kadziolka K, Litré F, Rousseaux P (2013). Combined treatment of brain AVMs with use of Onyx embolization followed by radiosurgery. AJNR Am J Neuroradiol.

[CR22] Pollock BE, Flickinger JC (2002). A proposed radiosurgery-based grading system for arteriovenous malformations. J Neurosurg.

[CR23] Reitz M, von Spreckelsen N, Vettorazzi E, Burkhardt T, Grzyska U, Fiehler J (2016). Angioarchitectural risk factors for hemorrhage and clinical long-term outcome in pediatric patients with cerebral arteriovenous malformations. World Neurosurg.

[CR24] Russin J, Spetzler R (2014). Commentary: the ARUBA trial. Neurosurgery.

[CR25] Spetzler RF, Martin NA (1986). A proposed grading system for arteriovenous malformations. J Neurosurg.

[CR26] Stapf C, Mast H, Sciacca RR, Choi JH, Khaw AV, Connolly ES, Pile-Spellman J, Mohr JP (2006). Predictors of hemorrhage in patients with untreated brain arteriovenous malformation. Neurology.

[CR27] Vaidya S, Tozer KR, Chen J (2008). An overview of embolic agents. Semin Interv Radiol.

[CR28] Valavanis A, Yaşargil MG (1998). The endovascular treatment of brain arteriovenous malformations. Adv Tech Stand Neurosurg.

[CR29] van Rooij WJ, Jacobs S, Sluzewski M, van der Pol B, Beute GN, Sprengers ME (2012). Curative embolization of brain arteriovenous malformations with onyx: patient selection, embolization technique, and results. AJNR Am J Neuroradiol.

[CR30] Vinters HV, Galil KA, Lundie MJ, Kaufmann JC (1985). The histotoxicity of cyanoacrylates. A selective review. Neuroradiology.

[CR31] Westphal M, Grzyska U. Clinical significance of pedicle aneurysms on feeding vessels, especially those located in infratentorial arteriovenous malformations. J Neurosurg. 2000:995–1001. 10.3171/jns.2000.92.6.0995 10839261

[CR32] Young AM, Teo M, Martin SC, Phang I, Bhattacharya JJ, St George EJ (2015). The diagnosis and management of brain arteriovenous malformations in a single regional center. World Neurosurg.

